# Community-Based Psychosocial Treatment Has an Impact on Social Processing and Functional Outcome in Schizophrenia

**DOI:** 10.3389/fpsyt.2018.00247

**Published:** 2018-06-08

**Authors:** Eszter Varga, Szilvia Endre, Titusz Bugya, Tamás Tényi, Róbert Herold

**Affiliations:** ^1^Department of Psychiatry and Psychotherapy, Medical School, University of Pécs Pécs, Hungary; ^2^Department of Psychology, University of Pécs Pécs, Hungary; ^3^Department of Cartography and Geoinformatics, University of Pécs Pécs, Hungary

**Keywords:** schizophrenia, social cognition, social functioning, functional outcomes, community-based treatment, rehabilitation, psychosocial treatment

## Abstract

Schizophrenic patients have serious impairments in social cognition, which often persists after significant reduction in clinical symptoms. Community-based psychosocial treatments aim to recover social functioning for mentally ill individuals. Our aim was to examine prospective changes in social cognition and functional outcomes in two groups of schizophrenic patients involved in two forms of community-based psychosocial treatments namely case management (CM) and community-based club (CC) compared to a matched, treatment as usual (TAU) group of patients. We hypothesized that CC and CM groups would exhibit better functional and social cognitive outcomes after a 6-month long psychosocial treatment period. Seventy-five patients participated either in CC, CM or TAU. Both CC and CM took part in community-based psychosocial treatment programs including trainings, such as communication and assertiveness trainings. In addition, CC provided group therapeutic treatments and a continuously available day care where patients had the possibility to participate in various social interactions. All participants were in remission, and on maintenance antipsychotic treatment. Participants were assessed on all study variables at two time points: baseline and after 6 months with a battery of questionnaires that examined affective face perception, affective prosody perception, pragmatic language comprehension and ToM. Our results showed that functional outcomes improved significantly in the CC as well as in the CM groups, in contrast to the TAU group. While analyzing summary scores of social cognition, it was found that only the CC group increased its performance in social cognition. In addition, a significant between-group difference in social cognitive function was found after 6 months between the three groups, with the CC group performing best. When investigating associations between changes in social cognition and changes in functional outcomes during a 6-month long treatment period, we found significant correlations between the two variables both in the CC and in the CM groups. Based on our results, we suggest that a rich interpersonal network and social support have highly beneficial effects on social cognition and we would like to emphasize the necessity of offering community-based psychosocial treatments beside antipsychotic medications as early as possible as a crucial part of the complex therapy of schizophrenia.

## Introduction

Schizophrenia is still one of the most disabling disorders throughout the world. With a prevalence of approximately 1%, it is a serious economic and social burden on communities (1). Despite major improvements in antipsychotic medications over recent decades, significant reductions in symptoms still often leave persistent impairments in social functioning (2). A meta-analysis of Jääskeläinen et al. (3) found that if the recovery criteria included both clinical as well as social domains, the recovery rate after the first episode of psychosis was as low as one in seven. These results clearly show that functional outcomes are impaired in schizophrenia (4).

Several researches reported that functional outcomes in schizophrenia are affected by numerous factors, including clinical symptoms, functional capacity, social cognition and neurocognition [(5, 6), and for a recent review see (4)], nevertheless, the relationships among these factors are complex.

Regarding syptomes, negative symptomes appeared to interfere more with functional outcomes than positive ones (6–8). Functional capacity in relation to functional outcomes is a rapidly developing concept, which refers to the skills underlying functional success, such as the skills necessarry to work or live independently (9). Interestingly, a recent study found that employment in schizophrenia is associated more with better symptomatic remission and communication skills than with neurocognitive functions (10). Social cognition is a multidimensional function, which consists of several domains, including Theory of Mind (ToM), emotion processing, social perception and attributional style. It refers to “the mental operations that underlie social interactions, including perceiving, interpreting, and generating responses to the intentions, dispositions, and behaviors of others” (11). Social cognitive impairments are significant in schizophrenia and seem to be a stable trait that precedes as well as predicts the illness onset (12–16). Previous studies found that social cognition mediates a significant indirect relationship between neurocognition and functional outcomes (17, 18) in schizophrenic patients. Emotion perception (17, 19), ToM (20), and social perception (21–23) in particular have shown promise to mediate the relationship between neurocognition and functional outcomes. Additionally, in a meta-analysis (18), social cognition was found to have a stronger impact on variance in community outcomes (16%) than neurocognition (6%).

Considering its functional importance, social cognition is a major treatment target in schizophrenia. Antipsychotic medications were found to be ineffective in significantly improving social cognition (24). At the same time, several psychosocial treatment programs have been developed, specifically targeting social cognitive functions (25). These programs are devoted to the enhancement of several aspect of social cognition (e.g., theory of mind, social cue perception, emotion recognition and regulation, etc.). A recent review of 16 controlled studies found moderate to large effect on several proximal measures (e.g., facial affect identification, theory of mind, social perception), and a modest but significant effect on more distal measures (e.g., general and negative symptoms) and recommended the broader use of social cognitive trainings (26). However, according to Horan and Green (25), findings about meaningful improvements in social functioning in patients treated with psychosocial interventions support a cautiously optimistic interpretation of these methods. It has also been found that integrating social cognitive treatment programs with psychosocial rehabilitation practices enhance functional outcomes, because patients are given opportunities to practice the learned skills in real-world social settings (27).

However, specific social cognitive treatments are still not widespread, and they are available only for a minority of patients. Still, a body of research suggests that psychosocial rehabilitation interventions may improve social functioning (4, 28), even though they don't target social cognition directly (26). Psychosocial rehabilitation practices are closely related to the concept of personal recovery as they have been developed “to help individuals with complex, long term mental health problems to develop the emotional, social and practical skills needed to live, learn and work in the community with the least amount of professional support” (29).

Community-based psychosocial treatment is a widely accepted form of psychosocial rehabilitation practice, the availability of which has increased in the past three decades in European countries (30–34). Community-based psychosocial treatment has been designed to support and improve community functioning as well as reduce relapse and hospitalization (35). Brekke et al. (28) reported a relationship between changes in psychosocial functioning and social cognition during community based psychosocial rehabilitation, and greater service intensity was related to higher level improvement in functioning.

In the present study, we examined the influence of two forms of community-based psychosocial treatment on social cognition, namely case management (CM) and community-based club (CC) (36), differing in service intensity and complexity. The basic approach of community-based psychosocial treatment is that resocialization can only be effective in a community where rehabilitation reduces stigmatization, motivates the patient and supports the family (37).

In case management (CM) model, the role of the case managers is to develop individualized care plans, together with the patients, in order to improve psychosocial strategies. Case managers also help patients cope with the difficulties of everyday life, such as shopping, paying bills or finding work. This type of support is crucial for patients and helps them develop coping mechanisms to deal with the seemingly unsolvable challenges of daily life. In order to help patients to acquire useful strategies, case managers also provide various trainings for the patients with the participation of family members or friends in the patients' homes. The long-term goal of the CM is to develop and maintain skills to cope with the difficulties of everyday life, improving the indices of relapses as well as patients' social functioning and standard of living (38, 39).

Community-based clubs (CC) are day care services (40), which have been formed based on the concept of the Clubhouse Model of psychosocial rehabilitation (41). Their main purpose is to help mentally ill people, usually living in social isolation, find their way to the community. Clubs are non-clinical communities composed of people with chronic psychiatric disorders over the age of 18, who are in the phase of stabilization or remission and staff who are active in club activities. A continuously available day care is provided for the patients where they have the possibility to participate in various social interactions and community programs, including various cultural programs and free time activities. Patients are also involved in various psychosocial interventions in a group format, including psychoeducation, social skills, communication as well as assertiveness trainings.

The aim of our study was to examine prospective changes in social cognition and functional outcomes in two groups of patients with schizophrenia involved in the two forms of community-based psychosocial treatments (namely CM and CC) compared to an age, gender, education and duration of illness matched, treatment as usual (TAU) group of schizophrenic patients. Members of the TAU group did not receive any psychosocial treatments. Patients were followed for 6 months. Based on previous findings of Brekke et al. (28), we hypothesized that CC and CM groups would exhibit better functional and social cognitive outcomes after a 6-month long psychosocial treatment period. Our further aim was to examine possible associations between changes in social cognition and changes in functional outcomes during the 6-month long treatment period. We assumed that significant correlations would be present between the two in the CM as well as in the CC groups.

## Materials and methods

### Participants

A total of 75 patients with schizophrenia (37 females and 38 males) fulfilling the diagnostic criteria of DSM-5 were evaluated. Diagnosis was confirmed by Module B and C of SCID-5 (Module B: Psychotic Symptoms, Module C: Differential Diagnosis of Psychotic Disorders) (42).

After a complete description of the study to the subjects, written informed consents were obtained. The investigation was done following institutional guidelines. Ethical perspectives were established in accordance with the latest version of the Declaration of Helsinki. The study design was approved by the Research Ethics Committee of the Faculty of Humanities, University of Pécs (2015/1). Patients were aware of the study aims and hypotheses.

Members of the CC and CM groups were recruited from Community-based Mental Health Care Institution Pécs, Hungary and members of the TAU group were recruited from Psychiatry Care Institution Pécs, Hungary. These are two separate institutions located in different parts of the city. All participants were outpatients and lived in their own homes. Patients were on maintenance antipsychotic treatment. Necessary conditions for participation were the following: age older than 18; native Hungarian speaker; no auditory or visual impairments that could cause difficulties in carrying out a computer test.; no evidence of substance abuse, no neurological disorder or mental retardation or cognitive deficits unrelated to schizophrenia; no changes in the medication of the participants during the study and in the last 6 months prior to the study; to be in the remission phase of the disease according to the remission criteria of schizophrenia (43).

### Treatments

A total of 75 schizophrenic patients took part in the study. We randomly assigned them into three groups, 26 (13 females and 13 males) took part in the community-based club treatment program (CC group), 26 (13 females and 13 males) took part in the case management treatment program (CM group) and 23 patients (11 females and 12 males) received treatment as usual (TAU group).

No participants left the study over the 6 months and all members of the CC and the CM group continued their rehabilitation treatment after the study protocol. After the study, community-based rehabilitation treatment was offered to members of the TAU group.

Age, gender, education, and duration of illness of the three groups were matched at baseline (Table 1).

**Table 1:** Demographic data and PANSS total remission score in the CC, the CM and the TAU.

**Variable**	**CC (*****n*** = **26)**			**CM (*****n*** = **26)**			**TAU (*****n*** = **23)**			**Between-group difference**	
	**Baseline**	**Post-treatment**	**Within-group difference**	**Baseline**	**Post-treatment**	**Within-group difference**	**Baseline**	**Post-treatment**	**Within-group difference**	**Baseline**	**Post-treatment**
	**Mean (S.D.)**	**Mean (S.D.)**	***p*****-value**^a^^,^^b^	**Mean (S.D.)**	**Mean (S.D.)**	***p*****-value**^a^^,^^b^	**Mean (S.D.)**	**Mean (S.D.)**	***p*****-value**^a^^,^^b^	***p*****-value**^a^^,^^c^	
Gender (male/female)	13/13	13/13	12/11								
Age (years)	39.6 (8.4)			37.5 (9.4)			39.9 (8.8)			0.159	
Education	3.7 (0.5)			3.6 (0.6)			3.9 (0.2)			0.157	
Duration of illness (years)	13.5 (5.6)			14.9 (5.9)			16.5 (6.5)			0.073	
PANSS total remission score	14.46 (2.02)	13.96 (1.98)	0.133	15.76 (2.88)	15.15 (3.27)	0.143	14.82 (2.2)	15.39 (2.29)	0.233	0.229	0.092

a *Statistically significant differences, two-tailed p < 0.05, uncorrected*.

b *Wilcoxon Signed-Rank Test was used for comparison within-groups*.

c *Kruskal–Wallis non-parametric test was used for comparison between groups*.

All three groups (CC, CM, and TAU) received antipsychotic pharmacotherapy and attended monthly consultations with their psychiatrist. In addition, both CC and CM took part in psychosocial treatment programs of the Community-based Mental Health Care Institution of Pécs, Hungary. Both treatment programs have been developed based on the latest Hungarian professional guidelines and standards. The TAU group did not receive any psychosocial treatments.

Members of the CC group were involved in a range of trainings and group activities, as well as in a continuously available day care in the community-based club, where they had the opportunity to participate in various social interactions with healthy as well as mentally ill members of the club. Patients took part in the following programs four times a week: psychoeducation, social skills, communication as well as assertiveness trainings, stress management, literature group, lifestyle group and music group. All the programs for the CC group took place in the club of the Community-based Mental Health Care Institution Pécs, Hungary.

Members of the CM group took part in the case management treatment program of the Community-based Mental Health Care Institution Pécs, Hungary. The program provided a personalized, community-based care in the patients' homes, assessing the deficiencies of the patients. Every patient had a highly personalized relationship with a case manager. The case manager visited the patient in his/her home once a week. The case manager developed an individualized care program, with the help of the patient, in which they defined the key problem areas of the patient's everyday social life. The case manager also helped the patient manage everyday tasks (shopping, cooking, cleaning, paying bills etc.), resolve crises and conflicts, and acquire useful strategies by providing various trainings (communication training, assertiveness trainings, developing problem-solving skills), held in the patient's home. Family members were also included in the trainings. The key element of the treatment was to enhance the psychosocial resources of the patient to better adapt to everyday life.

### Measures

Participants were assessed on all study variables at two time points: baseline and after 6 months. Assessments were completed by trained researchers.

#### Psychopathology and psychosocial functioning

We obtained data for psychopathology at baseline and after 6 months to confirm the remission state of the patients (Positive and Negative Syndrome Scale; PANSS). It was assessed with 8 items in positive, negative and general psychopathology subscales of PANSS (P1, P2, P3, N1, N4, N6, G5, G9), which were mild or less (≤3) for at least 6 months before entering the study as well as over the 6-month treatment period, according to the remission criteria of schizophrenia (43). Global Assessment of Functioning (GAF) Scale (44) was used to assess functionality. PANSS remission scores and GAF scores were assessed by two trained senior psychiatrists (Herold R, Tényi T). Inter-rater reliability was tested, and the kappa coefficient was >0.75 for all PANSS items and GAF scores.

#### Social cognition

Participants' assessments of social cognitive functions were carried out individually in separate examination rooms in the therapeutic institute through which they had been recruited. To assess social cognition, a newly developed psychometric software was used, called SCAN (Social Cognition Analyzer Application) (45, 46) in order to make the testing procedure more objective and reliable. Testing procedures were carried out by a trained administrator (Endre Sz) at both time points of testing. SCAN is a menu-driven application with a standard graphical interactive interface. It has a user-friendly mouse management, so the respondent can complete the test independently, after getting instructions. The test operator has no other job than to start the program and to do a backup of the recorded results after the test is completed. The English version of the software and its detailed manual with the social cognitive test battery can be downloaded from our website: scan.ttk.pte.hu.

At the time of testing, the program ran on a laptop (with 15′′ screen). The investigation was supported by a headset and a computer mouse attached to the laptop. SCAN is able to record response rates and response times. The results of each participant's test sessions are stored in separate folders, named after the respondent. Results from these folders can be imported into a spreadsheet for further analysis.

We used five experimental conditions: faux pas, irony, metaphor, emotion perception from faces and emotion perception from prosody. We presented 5 scenarios in the faux pas condition, 5 scenarios in the irony condition, 5 scenarios in the metaphor condition, 6 tasks in the emotion perception from faces condition and 24 tasks in the emotion perception from prosody condition, summing up to a total of 45 tasks in our study. In order to avoid learning, two different sets of task battery were used in each assessment. For each assessment, tasks were randomly selected from test materials of previous studies (47–53) that are described in the following paragraphs. There were no overlaps between the tasks in the assessments.

ToM was measured with Faux Pas (FP) tasks (47). Similar to our previous study (54), two questions were used in our adaptation to decrease the complexity of the task. The first question was about the recognition of FP situations and the second question was a false belief question.

As social inferencing in language pragmatics is an important aspect of social cognition (55), metaphor and irony tasks were used in order to examine pragmatic functions in detail. The metaphor and irony tasks were adopted from our previous studies (48–51). After each scenario two questions were asked about the figurative meaning of metaphors as well as two questions about the comprehension of ironic remarks in social situations. In the FP as well as in the irony and metaphor conditions two different sets of scenarios were used at baseline and after 6 months.

Emotion perception was measured with a reduced version of Face Test (52) and with an affective prosody test, which was designed based on Edwards et al. (53). An actress and an actor were asked to speak 24 (6 × 4) simple sentences with the appropriate affective prosody: “they must stay here”; “he will come soon”; “she will drive fast”; and “we must go there,” in the 6 basic moods in Hungarian. A total of 48 sentences were recorded. Twenty-four sentences were presented at baseline and twenty-four 6 months later. In both emotion perception tests, the perception of the six basic emotions were examined: anger, sadness, happiness, disgust, fear, and surprise. Participants had to choose from two possible answers (i.e., basic emotions) which best described the feelings seen or heard. For the next evaluation, 6 months later, a different set of pictures and sentences were used.

### Statistical analysis

We used Statistical Package for the Social Sciences [spss; SPSS Inc., Chicago, IL, USA; (56)] version 20 for Windows as well as OpenOffice.org version 5.0 to do the statistical analysis and to draw line charts. The distribution of data was checked with Kolmogorov-Smirnov goodness of fit. As distributions did not prove to be normal, Kruskal-Wallis one-way analysis of variance by ranks was performed to compare group medians across variables at baseline and after 6 months. The differences between each assessment were compared by group with Wilcoxon Signed-Rank Test.

For the correlation analysis, we used summary scores for the social cognition variable, because we were interested in global, rather than specific effects in these analyses (28). Summary social cognition score of each participant was determined by calculating the average percentage of all the results achieved in the five experimental conditions. Changes in GAF scores as well as in summary social cognition scores between baseline and after 6 months were calculated, with the extraction of the 6 months scores from the baseline scores in the three groups separately. After this method, Spearman's rank correlation coefficients (ρ) were calculated in the three groups to assess the relations between changes in GAF scores as well as changes in summary social cognition scores during the 6-month long treatment period.

## Results

### Demographic characteristics and psychosocial functioning

As gender, education, age, and duration of illness were matched between the three groups, there were no significant differences between them at baseline (Table 1).

At baseline the mean value of PANSS total remission score was 14.46 (2.02 S.D.) in the CC group, 15.76 (2.88 S.D.) in the CM group and 14.82 (2.02 S.D.) in the TAU group. After 6 months it was 13.96 (1.98 S.D.) in the CC group, 15.15 (3.27 S.D.) in the CM group and 15.39 (2.29 S.D.) in the TAU group. All the patients remained in remission during the 6 months. No significant symptom worsening, relapse or hospitalization were detected during the observation.

We found significant between-group differences in GAF scores at baseline (*p* = 0.001) as well as after 6 months (*p* < 0.001). In addition, compared to baseline GAF scores increased significantly after 6 months both in the CC group (*p* < 0.001) and in the CM group (*p* < 0.001; Table 2).

**Table 2:** Differences in GAF scores, social cognition task performance (%) and response time (sec) between CC, CM, and TAU groups at baseline compared to post-treatment.

	**CC group**			**CM group**			**TAU group**			**Between-group Difference**	
	**Baseline**	**Post-treatment**	**Within-group difference**	**Baseline**	**Post-treatment**	**Within-group difference**	**Baseline**	**Post-treatment**	**Within-group difference**	**Baseline**	**Post-treatment**
	**Mean (S.D.)**	**Mean (S.D.)**	***p*****-value**^a^^,^^c^	**Mean (S.D.)**	**Mean (S.D.)**	***p*****-value**^a^^,^^c^	**Mean (S.D.)**	**Mean (S.D.)**	***p*****-value**^a^^,^^c^	***p*****-value**^a^^,^^b^	
**GAF scores**	51.61 (1.32)	58.38 (1.76)	***<0.001***	50.34 (2.01)	52.19 (1.81)	***<0.001***	48.34 (1.64)	48.86 (1.21)	0.103	***0.001***	***0.001***
**Summary social cognition**	71.89 (11.37)	80.26 (9.94)	***<0.000***	74.66 (9.99)	77.21 (8.67)	0.469	74.26 (5.65)	75.29 (8.26)	0.393	0.373	**0.041**
Faux pas	79.80 (20.83)	79.61 (15.80)	0.703	83.76 (20.89)	79.23 (10.55)	0.124	90.32 (19.94)	77.39 (11.76)	**0.03**	0.060	0.165
Irony	58.84 (12.10)	79.32 (12.71)	***<0.000***	59.61 (10.76)	76.92 (16.84)	**0.01**	59.56 (9.28)	79.34 (12.84)	***0.001***	0.996	0.589
Metaphor	61.53 (25.40)	76.15 (16.26)	**0.031**	60.38 (21.99)	68.84 (21.60)	**0.033**	50.43 (18.45)	61.30 (18.41)	0.120	0.183	**0.01**
Face test	82.69 (17.93)	80.76 (16.11)	0.667	89.74 (13.39)	75.64 (17.14)	**0.012**	88.40 (18.23)	73.18 (18.62)	**0.016**	0.295	0.062
Emotional prosody	76.60 (11.79)	85.45 (11.72)	***0.001***	80.12 (14.54)	85.45 (8.89)	0.166	82.60 (10.48)	85.25 (6.11)	0.808	0.166	0.313
**Summary response time**	8.51 (3.19)	8.11 (3.09)	0.694	9.26 (3.87)	8.79 (3.32)	0.534	6.91 (2.08)	8.31 (3.02)	0.101	0.055	0.565
Faux pas	8.01 (3.61)	8 (4.25)	0.732	9.19 (9.85)	7.54 (2.97)	0.517	7.89 (3.5)	8.47 (3.09)	0.394	0.980	0.253
Irony	6.6 (2.34)	7.35 (2.19)	0.066	7.12 (2.66)	10.35 (4.78)	***0.005***	6.2 (2.6)	8.81 (3.2)	**0.011**	0.505	***<0.000***
Metaphor	13.06 (6.56)	9.35 (3.39)	**0.019**	14.23 (6.01)	11.33 (6.3)	0.052	9.71 (3.77)	8.97 (3.18)	0.249	**0.02**	0.052
Face test	8.93 (3.65)	10.69 (10.58)	0.354	10.04 (5)	9.76 (5.10)	0.879	6.43 (2.17)	10.76 (12.54)	***<0.000***	***0.005***	0.695
Emotional prosody	5.95 (2.72)	5.18 (1.68)	0.485	5.72 (2.4)	4.95 (1.6)	0.282	4.33 (1.43)	4.54 (1.08)	0.627	**0.043**	0.550

a Wilcoxon Signed-Rank Test was used for comparing response accuracy within-groups.

b *Kruskal–Wallis non-parametric test was used for comparing response accuracy between groups*.

c *Values in bold presents statistically significant differences, two-tailed p < 0.05, uncorrected. Values in italic present significant differences after Bonferroni correction (p < 0.01)*.

### Social cognition task performance and response time

In the within-group analysis of the summary social cognitive scores the CC group was found to have increased their performance significantly (*p* < 0.001), while in the between-group analysis significant differences were found between the three groups at 6 months (*p* = 0.041). The last significant difference between the three groups disappeared after Bonferroni correction. No statistical differences were found regarding the summary response time during the social cognitive tasks between the three groups at baseline or after 6 months.

While analyzing the data of social cognition task performance in details, in the within-group analysis task performance of the *CC group* increased significantly in the irony task (*p* < 0.001), in the metaphor task (*p* = 0.031) and in the emotional prosody task (*p* = 0.001). The *CM group* also showed better results in the irony task (*p* = 0.01) and in the metaphor task (*p* = 0.033), however, they showed worse results in the face test (*p* = 0.012). Interestingly, the *TAU group* showed better performance only in the irony task (*p* = 0.001), and their performance in FP task (*p* = 0.03), as well as in face perception (*p* = 0.016) deteriorated significantly.

However, significant improvement of the CC in the metaphor task, and significant improvement of the CM in the irony and in the metaphor task disappeared after Bonferroni correction. The significant decline of the TAU in the FP task and in the face test as well as significant decline of the CM in the face test also disappeared after Bonferroni correction.

In the between-group comparison, there were no significant differences regarding any of the task performances at baseline. The evaluations 6 months later showed significant differences in the metaphor tasks (*p* = 0.01), but this significant difference between the three groups disappeared after Bonferroni correction.

While analyzing response times of social cognition tasks in detail in the within group differences, the *CC group* showed significant acceleration in the metaphor tasks (*p* = 0.019). Interestingly, the *CM group* showed deceleration in the irony task (*p* = 0.005), while the *TAU group* also showed deceleration in the irony task (*p* = 0.011) as well as in the face test (*p* < 0.001) between baseline and after 6 months.

After the Bonferroni correction, the acceleration in the metaphor task in the CC group, as well as the deceleration in the irony task in the TAU group ceased to be significant.

On the between group-level, at baseline, the three groups differed significantly in their response times in the metaphor task (*p* < 0.001) and in the face test (*p* = 0.005). Besides, response time in the irony task remained significant (*p* = 0.01) after 6 months. However, the latter significant difference between the three groups disappeared after Bonferroni correction.

**Figure 1:**
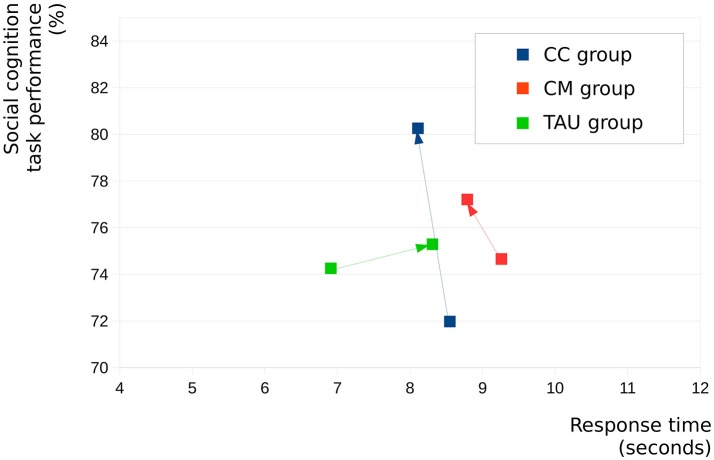
Changes in summary social cognition scores and summary response times between baseline and after 6 months in the three groups separately.

Figure 1 graphically shows changes in summary social cognition as well as summary response times between baseline and after 6 months in the three groups separately. It shows that among the three groups the CC group reached the highest response rate (significant) together with the fastest response time (not significant) in social cognitive tasks 6 months after baseline.

### Correlations

We found significant positive correlations between changes in GAF scores and changes in summary social cognition scores during the 6-month long treatment period in the CC group (*r* = 0.414, *p* = 0.035) as well as in the CM group (*r* = 0.416, *p* = 0.034). However, in the TAU group this correlation was not significant (*r* = −0.122, *p* = 0.579).

## Discussion

In the present research our aim was to examine prospective changes over a 6-month long treatment period, in social cognition and functional outcomes in two groups of schizophrenic patients in remission, involved in two forms of community-based psychosocial treatments compared to a matched treatment as usual group of schizophrenic patients. Our results showed that functional outcomes improved significantly in the CC as well as in the CM groups, in contrast to the TAU group. While analyzing summary scores of social cognition, it was found that only the CC group increased its performance in social cognition. In addition, a significant between-group difference in social cognitive function was found after 6 months between the three groups, with the CC group performing at the highest rate. Our second aim was to examine possible associations between changes in social cognition and changes in functional outcomes during a 6-month long treatment period, and we found significant correlations between the two variables both in the CC as well as in the CM groups. In line with previous investigations (28, 57), we found, that both community-based psychosocial treatment programs improve functional outcomes in schizophrenia.

In the present research, all participants were in the remission phase of their disease, based on the operationalized criteria of the Remission in Schizophrenia Working Group (RSWG) (43). Since symptomatic resolution often leaves serious impairments in social functioning (2), there is a need to shift researchers' attention beyond remission to a more complex concept of recovery. There is still a lack of consensus on the definition of recovery, but there have been suggestions that at least two areas should be considered: clinical remission and social functioning (4). Schennach-Wolff et al., (2) proposed that for complete remission both clinical and functional remissions should be taken into consideration. In the present study, GAF scores were used to measure functional outcomes. If functional remission was defined as GAF score being above 61 (2), four participants from the CC group (15%) would have reached the status of functional remission with achieving higher than 61 points in GAF. However, these results should be interpreted cautiously since in addition to GAF scores, in the literature other metrics are often used to measure functional outcomes in detail (2, 58). On the other hand, it should be kept in mind, that the outcome was examined after a treatment period of 6 months, which may be a time period too short for schizophrenic patients to achieve functional remission. Interestingly, no participants from the CM or the TAU groups were able to reach such functional remission.

We found modest positive correlations between prospective changes in summary social cognition scores and functional outcomes both in the CC as well as in the CM groups. However, there was no such relationship observed in the TAU group. In the biosocial model of functional outcomes in schizophrenia (19), social cognition (measured with emotion perception tasks) was found to have both direct and indirect influence on functional outcomes. In a more detailed research, Hoe et al. (59) found that neurocognition is causally primary to social cognition, and that neurocognition and social cognition are causally primary to functional outcome. In line with our present finding, Adamczik et al. (10) have found, that functional capacity was influenced more by communication skills (i.e., humor, metaphor, emotional and linguistic prosody) than by neurocognitive functions. Based on the findings of these studies, we can only speculate that in the present study improvements in social cognition was primary to improvements in functional outcomes in patients involved in the CC and the CM groups.

The most prominent changes in social cognitive functions were detected in the *CC group*, as they showed strong significant prospective changes in irony comprehension, emotional prosody processing and summary social cognition, as well as a moderate prospective improvement in metaphor comprehension. Moreover, a significant between-group difference was found after 6 months in the metaphor tasks with the CC group performing at the highest rate. Importantly, the CC group also significantly reduced their response times in the metaphor tasks. According to our previous study (45), for adaptive social functioning the correct interpretation of the social stimuli is as important as processing it in a timely manner.

Regarding the results of the CC group, we propose that with a sufficiently effective psychosocial rehabilitation program, patients with schizophrenia are able to meaningfully improve their social cognitive performance. In line with Medalia and Saperstein (27) we propose that this improvement was due to the basic approach of the community-based club: patients were not only involved in several trainings and group activities in order to improve their social functioning, but a continuous day care was also available for them, where they had the possibility to participate in various social interactions with other members of the club. In this social milieu patients are given opportunities to practice social skills in real-world settings, providing an aspecific effect to their social cognitive abilities. We use the term aspecific, because we did not use specific social cognitive interventions targeting social cognitive abilities, and we can only speculate that the improving effect appeared due to the supportive social milieu, and the various social interactions patients could participate in. We also propose that being involved in a therapeutic community may support finding new social roles for patients, which may also help to decrease feeling stigmatized as well as increase the chance for resocialization into the real-world community. Our results emphasize the role of environmental stimuli. According to Bora and Murray (60) the decreased environmental stimuli related to social isolation may play a role even in brain volume reduction associated with schizophrenia. The richness of interpersonal networks and social support can be conceived of as a protective factor that may facilitate coping mechanisms and competence, counterbalancing the unfavorable effects of social stresses (61, 62). Our results are in line with the suggestions of recovery-oriented services, which emphasize the role of peer relationships, and social networks in the recovery process (63).

Regarding the CM group, we observed moderate prospective improvements in irony and metaphor comprehension, together with a moderate decline in affective face perception. Additionally, there was a significant deceleration of response time in irony comprehension. Our results also show that the TAU group significantly improved response rates, together with a moderate deceleration in response times, in the irony tasks. Their performance also moderately declined in the faux pas tasks as well as in the face test. Regarding the differences observed between the three groups after 6 months of treatment, it can be observed, that the TAU group had the lowest scores in summary social cognition, in the metaphor tasks, and in the GAF scale. In addition, their performance was the slowest in the irony tasks.

We cannot ignore the fact that all three groups improved their performance significantly in the irony tasks. Additionally, both the CM and the TAU increased their response times, which resulted in a significant between-group difference in response times of the irony tasks after 6 months treatment. In Varga et al. (45) we have found that healthy participants perform irony tasks significantly faster (Mean: 3.5; S.D.: 1.3) than schizophrenic patients (Mean: 6.8; S.D.: 5.2), implying that for a non-clinical population, irony comprehension is a fast process. In linguistic studies it has been found that the processing of irony is faster if it does not require activating the literal meaning of its components, compared to those in which priming irrelevant meanings would result in slower processing times (64). In Gyori et al. (55), a possible mechanism for compensating irony comprehension was investigated, called the “reality-based shortcut strategy,” in which activating both literal and non-literal meaning of the utterance is required. In our previous investigations, we have assumed that both schizophrenic patients with higher IQ (50) and first-degree relatives of schizophrenic patients (51) might use similar strategies to perform irony tasks. In the present study, we can only speculate that elongated response times of the CM and the TAU in the irony tasks were the results of such compensatory strategy.

## Conclusions

As far as we know, the present investigation is the first to assess the efficacy of two forms of community-based psychosocial treatments available for schizophrenic patients and compared them to a treatment as usual group of patients. According to our present findings, we believe that community-based psychosocial treatments are able to facilitate functional outcomes in schizophrenia. Regarding social cognition, we found that the most prominent prospective change could be achieved with the program of community-based clubs. Our results indicate that this is due to a supportive social milieu, in which various social interactions can be practiced as well as new social roles can be learned, which provide aspecific improving effect to social cognition and help patients find their way to the society. Additionally, our present results clearly show that in order to meaningfully improve the social cognitive performance of patients, it is important for them to get involved in community-based clubs. According to our present and previous (45) findings and the biosocial model of functional outcomes in schizophrenia (19), we propose that social cognitive treatments, for example metacognitive training (MCT) (65, 66), cognitive remediation programs, or interventions targeting communication abilities (10) used in conjunction with community-based psychosocial rehabilitation programs would further facilitate functional outcomes in schizophrenia.

To summarize, our results underline the necessity of offering community-based psychosocial treatments beside antipsychotic pharmacological treatments (33, 67). Community-based psychosocial treatments supporting the richness of interpersonal network and social support is a crucial part of the complex therapy of schizophrenia.

## Limitations

Our study has several limitations. First of all, there is a wide range of community-based psychosocial treatment models in the literature, and the generalization of our present results across different models could be problematic. However, as far as we know, our research was the first that examined different aspects of community-based rehabilitation separately.

As we mentioned earlier we used only GAF for estimating functionality, and a more detailed assessment of these domains would have eventuated in more reliable results. We should have also considered other areas referring to functionality such as self-care, independent living, work or supported work, etc.

We did not assess potential relations between neurocognitive factors, social cognition, functional outcomes, clinical symptoms and vocational status. Future studies should address the effects between these factors. In addition, other aspects of social cognition such as attributional style as well as social perception should be reflected in future measures.

Another important limitation is the use of different antipsychotic medications. All patients with schizophrenia were on maintenance antipsychotic medication. However, it was found that antipsychotics have little reliable effect on social cognition (24), and earlier studies did not find any correlation between social cognition and antipsychotic treatment (68).

## Author contributions

EV: study design, stimulus construction, data analysis, writing article, manuscript revision; SE: project idea, study design, data collection, data analysis, writing article; TB: writing software, software development, data analysis; TT: writing article, manuscript revision; RH: project idea, writing article, manuscript revision.

### Conflict of interest statement

The authors declare that the research was conducted in the absence of any commercial or financial relationships that could be construed as a potential conflict of interest.

## References

[1] Mueser KT Bond GR Drake RE Resnick SG. Models of community care for severe mental illness: a review of research on case management. Schizophr Bull. (1998) **24**:37. 9502546 10.1093/oxfordjournals.schbul.a033314

[2] Schennach-Wolff R Jäger M Seemüller F Obermeier M Messer T Laux G Pfeiffer H. Defining and predicting functional outcome in schizophrenia and schizophrenia spectrum disorders. Schizophr Res. (2009) 113:210–7. 10.1016/j.schres.2009.05.032 19560901

[3] Jääskeläinen E Juola P Hirvonen N McGrath JJ Saha S Isohanni M Veijola J. A systematic review and meta-analysis of recovery in schizophrenia. Schizophr Bull. (2012) 39:1296–306. 10.1093/schbul/sbs130 23172003 PMC3796077

[4] Morin L Franck N. Rehabilitation Interventions to Promote Recovery from Schizophrenia: a Systematic Review. Front Psychiatry (2017) **8**:100. 10.3389/fpsyt.2017.00100 28659832 PMC5467004

[5] Galderisi S Rossi A Rocca P Bertolino A Mucci A Bucci P. The influence of illness-related variables, personal resources and context-related factors on real-life functioning of people with schizophrenia. World Psychiatry (2014) 13:275–87. 10.1002/wps.20167 25273301 PMC4219069

[6] Bliksted V Videbech P Fagerlund B Frith C. The effect of positive symptoms on social cognition in first-episode schizophrenia is modified by the presence of negative symptoms. Neuropsychology (2017) **31**:209. 10.1037/neu0000309 27808537

[7] Lin CH Huang CL Chang YC Chen PW Lin CY Tsai GE Lane HY. Clinical symptoms, mainly negative symptoms, mediate the influence of neurocognition and social cognition on functional outcome of schizophrenia. Schizophr Res. (2013) 146:231–7. 10.1016/j.schres.2013.02.009 23478155

[8] Ventura J Hellemann GS Thames AD Koellner V Nuechterlein KH. Symptoms as mediators of the relationship between neurocognition and functional outcome in schizophrenia: a meta-analysis. Schizophr Res. (2009) 113:189–99. 10.1016/j.schres.2009.03.035 19628375 PMC2825750

[9] Harvey PD Strassing M. Predicting the severity of everyday functional disability in people with schizophrenia: cognitive deficits, functional capacity, symptoms, and health status. World Psychiatry (2012) 11:73–9. 10.1016/j.wpsyc.2012.05.004 22654932 PMC3363376

[10] Adamczyk P Daren A Sułecka A Bładzinski P Cichocki Ł Kalisz A. Do better communication skills promote sheltered employment in schizophrenia?. Schizophr Res. (2016) 176:331–9. 10.1016/j.schres.2016.08.015 27546092

[11] Green MF Penn DL Bentall R Carpenter WT Gaebel W Gur RC. Social cognition in schizophrenia: an NIMH workshop on definitions, assessment, and research opportunities. Schizophr Bull. (2008) 34:1211–20. 10.1093/schbul/sbm145 18184635 PMC2632490

[12] Frith CD Corcoran R. Exploring ‘theory of mind’ in people with schizophrenia. Psychol Med. (1996) 26:521–30. 10.1017/S0033291700035601 8733211

[13] Riveros R Manes F Hurtado E Escobar M Reyes MM Cetkovich M. Context-sensitive social cognition is impaired in schizophrenic patients and their healthy relatives. Schizophr Res. (2010) 116:297–8. 19914806 10.1016/j.schres.2009.10.017

[14] Savla GN Vella L Armstrong CC Penn DL Twamley EW. Deficits in domains of social cognition in schizophrenia: a meta-analysis of the empirical evidence. Schizophr Bull. (2012) 39:979–92. 10.1093/schbul/sbs080 22949733 PMC3756768

[15] Pinkham AE Penn DL Green MF Buck B Healey K Harvey PD. The social cognition psychometric evaluation study: results of the expert survey and RAND panel. Schizophr Bull. (2013) 40:813–23. 10.1093/schbul/sbt081 23728248 PMC4059426

[16] Green MF Horan WP Lee J Social cognition in schizophrenia. Nat Rev Neurosci. (2015) **16**:620. 10.1038/nrn4005 26373471

[17] Schmidt SJ Mueller DR Roder V. Social cognition as a mediator variable between neurocognition and functional outcome in schizophrenia: empirical review and new results by structural equation modeling. Schizophr Bull. (2011) 37(suppl. 2):S41–54. 10.1093/schbul/sbr079 21860046 PMC3160114

[18] Fett AK Viechtbauer W Penn DL van Os J Krabbendam L. The relationship between neurocognition and social cognition with functional outcomes in schizophrenia: a meta-analysis. Neurosci Biobehav Rev. (2011) 35:573–88. 10.1016/j.neubiorev.2010.07.001 20620163

[19] Brekke J Kay DD Lee KS Green MF. Biosocial pathways to functional outcome in schizophrenia. Schizophr Res. (2005) 80:213–25. 10.1016/j.schres.2005.07.008 16137859

[20] Couture SM Granholm EL Fish SC. A path model investigation of neurocognition, theory of mind, social competence, negative symptoms and real-world functioning in schizophrenia. Schizophr Res. (2011) 125:152–60. 10.1016/j.schres.2010.09.020 20965699 PMC3031755

[21] Addington J. Saeedi H Addington D. Influence of social perception and social knowledge on cognitive and social functioning in early psychosis. Br J Psychiatry (2006) 189:373–8. 10.1192/bjp.bp.105.021022 17012662

[22] Sergi MJ Rassovsky Y Nuechterlein KH Green MF. Social perception as a mediator of the influence of early visual processing on functional status in schizophrenia. Am J Psychiatry (2006) 163:448–54. 10.1176/appi.ajp.163.3.448 16513866

[23] Vauth R Rüsch N Wirtz M Corrigan PW. Does social cognition influence the relation between neurocognitive deficits and vocational functioning in schizophrenia?. Psychiatry Res. (2004) 128:155–65. 10.1016/j.psychres.2004.05.018 15488958

[24] Kucharska-Pietura K Mortimer A. Can antipsychotics improve social cognition in patients with schizophrenia?. CNS Drugs (2013) 27:335–43. 10.1007/s40263-013-0047-0 23533009 PMC3657085

[25] Horan WP Green MF. Treatment of social cognition in schizophrenia: Current status and future directions. Schizophr Res. (2017) 10.1016/j.schres.2017.07.013. [Epub ahead of print]. 28712968

[26] Kurtz MM Gagen E Rocha NB Machado S Penn DL. Comprehensive treatments for social cognitive deficits in schizophrenia: a critical review and effect-size analysis of controlled studies. Clin Psychol Rev. (2016) 43:80–9. 10.1016/j.cpr.2015.09.003 26437567

[27] Medalia A Saperstein AM. Does cognitive remediation for schizophrenia improve functional outcomes?. Curr Opin Psychiatry (2013) 26:151–7. 10.1097/YCO.0b013e32835dcbd4 23318663

[28] Brekke JS Hoe M Long J Green MF. How neurocognition and social cognition influence functional change during community-based psychosocial rehabilitation for individuals with schizophrenia. Schizophr Bull. (2007) 33:1247–56. 10.1093/schbul/sbl072 17255120 PMC2632359

[29] Bitter NA Roeg DP van Nieuwenhuizen C van Weeghel J. Effectiveness of the Comprehensive Approach to Rehabilitation (CARe) methodology: design of a cluster randomized controlled trial. BMC Psychiatry (2015) **15**:165. 10.1186/s12888-015-0564-0 26198855 PMC4510908

[30] Falloon IR Montero I Sungur M Mastroeni A Malm U Economou M. Implementation of evidence-based treatment for schizophrenic disorders: two-year outcome of an international field trial of optimal treatment. World Psychiatry (2004) 3:104–9. 16633471 PMC1414683

[31] Cechnicki A Bielanska A A community treatment programme for people suffering from schizophrenia in Krakow. In: Therapeutic Communities for Psychosis: Philosophy, History and Clinical Practice, J. Gale, A. Realpe, and E. Pedriali editors, New York, NY: Routledge (2008). p. 171–85.

[32] Cechnicki A Towards psychotherapy-oriented community psychiatry−30 years of experiences in Kraków. Arch Psychiatry Psychother. (2011) 1:71–80.

[33] Asher L Patel V De Silva MJ. Community-based psychosocial interventions for people with schizophrenia in low and middle-income countries: systematic review and meta-analysis. BMC Psychiatry (2017) **17**:355. 10.1186/s12888-017-1516-7 29084529 PMC5661919

[34] Bronowski P Sawicka M Rowicka M Jarmakowicz M. Social networks and social functioning level among occupational therapy workshops and community-based support centers users. Psychiatria Polska. (2017) **51**:139-52. 28455901 10.12740/PP/62080

[35] Brekke JS Long JD. Community-based psychosocial rehabilitation and prospective change in functional, clinical, and subjective experience variables in schizophrenia. Schizophr Bull. (2000) **26**:667. 10.1093/oxfordjournals.schbul.a033485 10993405

[36] Bugarszki Zs A közösségi pszichiátriai ellátásról. Esély (2006) 1:67–74.

[37] Mueser KT Deavers F Penn DL Cassisi JE. Psychosocial treatments for schizophrenia. Ann Rev Clin Psychol. (2013) 9:465–97. 10.1146/annurev-clinpsy-050212-185620 23330939

[38] Holloway F Carson J. Case management: an update. Int J Soc Psychiatry (2001) 47:21–31. 11589333 10.1177/002076400104700303

[39] Thornicroft G The concept of case management for long-termmental illness. Int Rev Psychiatry (1991) 3:125–32.

[40] Hultqvist J. Markström U. Tjörnstrand C. Eklund M Social networks and social interaction among people with psychiatric disabilities: comparison of users of day centres and clubhouses. Global J Health Sci. (2017) 9:107–20. 10.5539/gjhs.v9n6p107

[41] McKay C Nugent KL Johnsen M Eaton WW Lidz CW. A systematic review of evidence for the clubhouse model of psychosocial rehabilitation. Adm Policy Ment Health (2018) 45:28–47. 10.1007/s10488-016-0760-3 27580614 PMC5756274

[42] First MB Williams JBW Karg RS Spitzer RL: Structured Clinical Interview for DSM-5 Disorders, Clinician Version (SCID-5-CV). Arlington, VA: American Psychiatric Association (2015).

[43] Andreasen NC Carpenter WT Jr Kane JM Lasser RA Marder SR Weinberger DR. Remission in schizophrenia: proposed criteria and rationale for consensus. Am J Psychiatry (2005) 162:441–9. 10.1176/appi.ajp.162.3.441 15741458

[44] Jones SH Thornicroft G Coffey M Dunn G. A brief mental health outcome scale-reliability and validity of the Global Assessment of Functioning (GAF). Br J Psychiatry (1995) 166:654–9. 7620753 10.1192/bjp.166.5.654

[45] Varga E Bugya T Endre S Herold R Horváth R Mákos O. A new method for the measurement of social cognition in schizophrenia. Psychiatr Hung. (2017) 32:313–31. 29135445

[46] Varga E Herold R Tényi T Bugya T Social Cognition Analyzer Application (SCAN)-a new approach to analyse social cognition in schizophrenia. Eur Neuropsychopharmacol. (2018) 28:S55–6. 10.1016/j.euroneuro.2017.12.083

[47] Baron-Cohen S O'riordan M Stone V Jones R Plaisted K. Recognition of faux pas by normally developing children and children with Asperger syndrome or high-functioning autism. J Autism Dev Disord. (1999) 29:407–18. 10587887 10.1023/a:1023035012436

[48] Herold R Tényi T Lénárd K Trixler M. Theory of mind deficit in people with schizophrenia during remission. Psychological Med. (2002) 32:1125–9. 10.1017/S0033291702005433 12214792

[49] Varga E Simon M Tényi T Schnell Z Hajnal A Orsi G. Irony comprehension and context processing in schizophrenia during remission–A functional MRI study. Brain Lang. (2013) 126:231–42. 10.1016/j.bandl.2013.05.017 23867921

[50] Varga E Schnell Z Tényi T Németh N Simon M Hajnal A Compensatory effect of general cognitive skills on non-literal language processing in schizophrenia: a preliminary study. J Neurolinguist. (2014) 29:1–6. 10.1016/j.jneuroling.2014.01.001

[51] Herold R Varga E Hajnal A Hamvas E Berecz H Tóth B. Altered neural activity during irony comprehension in unaffected first-degree relatives of schizophrenia patients–an fMRI study. Front Psychol. (2017) **8**:2309. 10.3389/fpsyg.2017.02309 29375430 PMC5767266

[52] Baron-Cohen S Wheelwright S Jolliffe AT Is there a “language of the eyes”? Evidence from normal adults, and adults with autism or Asperger syndrome. Visual Cogn. (1997) 4:311–31. 10.1080/713756761

[53] Edwards J Pattison PE Jackson HJ Wales RJ. Facial affect and affective prosody recognition in first-episode schizophrenia. Schizophr Res. (2001) 48:235–53. 10.1016/S0920-9964(00)00099-2 11295377

[54] Herold R Feldmann A Simon M Tenyi T Kövér F Nagy F Varga E Fekete S. Regional gray matter reduction and theory of mind deficit in the early phase of schizophrenia: a voxel-based morphometric study. Acta Psychiatr Scand. (2009) 119:199–208. 10.1111/j.1600-0447.2008.01297.x 19016669

[55] Gyori M Lukács Á Pléh C Towards the understanding of the neurogenesis of social cognition: Evidence from impaired populations. J Cult Evol Psychol. (2004) 2:261–82. 10.1556/JCEP.2.2004.3-4.

[56] Nie NH Bent DH Hull CH SPSS: Statistical Package for the Social Sciences. New York, NY: McGraw-Hill (1970).

[57] Brekke JS Long JD Nesbitt N Sobel E. The impact of service characteristics on functional outcomes from community support programs for persons with schizophrenia: A growth curve analysis. J Consult Clin Psychol. (1997) **65**:464. 9170770 10.1037//0022-006x.65.3.464

[58] Wittorf A Wiedemann G Buchkremer G Klingberg S. Prediction of community outcome in schizophrenia 1 year after discharge from inpatient treatment. Eur Arch Psychiatry Clin Neurosci. (2007) 258:48–58. 10.1007/s00406-007-0761-z 17990052

[59] Hoe M Nakagami E Green MF Brekke JS. The causal relationships between neurocognition, social cognition and functional outcome over time in schizophrenia: a latent difference score approach. Psychol Med. (2012) 42:2287–99. 10.1017/S0033291712000578 22475159

[60] Bora E Murray RM. Meta-analysis of cognitive deficits in ultra-high risk to psychosis and first-episode psychosis: do the cognitive deficits progress over, or after, the onset of psychosis?. Schizophr Bull. (2013) 40:744–55. 10.1093/schbul/sbt085 23770934 PMC4059428

[61] Beels CC. Social support and schizophrenia. Schizophr Bull. (1981) 7:58–72. 10.1093/schbul/7.1.58 7233113

[62] Buchanan J. Social support and schizophrenia: a review of the literature. Arch Psychiatric Nursing (1995) 9:68–76. 10.1016/S0883-9417(95)80003-4 7755410

[63] Galletly C Castle D Dark F Humberstone V Jablensky A Killackey E. Royal Australian and New Zealand College of Psychiatrists clinical practice guidelines for the management of schizophrenia and related disorders. Aust N Z J Psychiatry (2016) 50:410–72. 10.1177/0004867416641195 27106681

[64] Giora R On Our Mind: Salience, Context, and Figurative Language. Oxford: Oxford University Press (2003).

[65] Van Oosterhout B Smit F Krabbendam L Castelein S Staring AB Van der Gaag M. Metacognitive training for schizophrenia spectrum patients: a meta-analysis on outcome studies. Psychol Med. (2016) 46:47–57. 10.1017/S0033291715001105 26190517

[66] Varga E Endre S Molnár D Tényi T Herold R Efficacy of metacognitive training compared with a psychosocial rehabilitation program on social cognitive processing in schizophrenia. Eur Neuropsychopharmacol. (2017) **27**:S959. 10.1016/S0924-977X(17)31693-0

[67] Tabo A Aydin E Yumrukçal H Yigit S Uzun UE Karamustafalioglu O. Longer duration of untreated psychosis hinders improvement in treatment of chronic schizophrenia: community based early intervention is an evidence based option. Commun Mental Health J. (2017) 53:929–35. 10.1007/s10597-017-0088-9 28188388

[68] Mo S Su Y Chan RC Liu J. Comprehension of metaphor and irony in schizophrenia during remission: the role of theory of mind and IQ. Psychiatry Res. (2008) 157:21–9. 10.1016/j.psychres.2006.04.002 17854910

